# MALDI-TOF MS Analysis of Native and Permethylated or Benzimidazole-Derivatized Polysaccharides 

**DOI:** 10.3390/molecules17054950

**Published:** 2012-04-30

**Authors:** Wei-Ting Hung, Shwu-Huey Wang, Yi-Ting Chen, Hui-Ming Yu, Chung-Hsuan Chen, Wen-Bin Yang

**Affiliations:** 1 Genomics Research Center, Academia Sinica, 128, Academia Road Sec. 2, Taipei 115, Taiwan; 2 Core Facility Center, Office of Research and Development, Taipei Medical University, 250 Wu-Hsing Street, Taipei 110, Taiwan

**Keywords:** polysaccharides, MALDI-TOF MS, permethylation, benzimidazole, 2,5-DHB

## Abstract

MALDI-TOF MS provides rapid and sensitive analyses of larger biomolecules. However, MS analyses of polysaccharide have been reported to have lower sensitivity compared to peptides and proteins. Here, we investigated some polysaccharides chemically derivatized by permethylation and *ortho*-phenylene diamine (OPD) tagging. Methylated glycan is obviously able to improve the sensitivity for mass spectrometry detection. Oxidative condensation by UV-activation tagging to saccharides by OPD and peptide-OPD also improve the sensitivity of MALDI-TOF MS analyses. Polysaccharides including dextran, glucomannan, arabinoxylan, arabinogalactan and beta-1,3-glucan, isolated from nutritional supplements of *Ganoderma lucidum* and *Saccharomyces pastorianus* were measured using MALDI-TOF MS with 2,5-dihydroxybenzoic acid (2,5-DHB) as the matrix. These glycans were also derivatized to methylated and benzimidazole-tagged glycans by chemical transformation for molecular weight analysis. The derivatized polysaccharides showed excellent MALDI-TOF MS signal enhancement in the molecular weight range from 1 to 5 kDa. Here, we demonstrate an efficient method to give glycan-benzimidazole (glycan-BIM) derivatives for polysaccharide determination in MALDI-TOF MS. Therefore, permethylated or benzimidazole-derivatized polysaccharides provide a new option for polysaccharide analysis using MALDI-TOF MS.

## 1. Introduction

Polysaccharides play essential roles in all living organisms. However, many saccharides cannot be clearly defined due to the difficulties in saccharide detection. Matrix-assisted laser desorption/ionization (MALDI) mass spectrometry (MS) has been successfully developed as a soft ionization method in MS detection for biopolymers and macromolecules [1]. However, compared with proteins or nucleotides with comparable molecular weights MS still struggles to measure large polysaccharides. Appropriate tags can be attached to saccharides to improve their ionization efficiency, and thus enhance the corresponding MS signals [2]. Most polysaccharides have huge molecular weights and show poor ionization efficiency, probably due to their poor capability in carrying charge for mass spectrometric detection. The MS technique is capable of the direct mass measurement of derivatized or underivatized oligosaccharides such as *N*- and *O*-glycans and low molecular weight glycans [3,4]. Mock and coworkers reported the first measurement of unmodified oligosaccharides with MALDI-TOF MS using 3-amino-4-hydroxybenzoic acid as the matrix [5]. Stahl and coworkers have subsequently discovered that 2,5-dihydroxybenzoic acid (2,5-DHB) is a better matrix for the MALDI-TOF MS measurement of saccharides [6]. Hsu *et al*. also found that 2',4',6'-trihydroxyacetophenone is a suitable matrix for producing the dominant molecular ions of saccharides [7]. Although MALDI-TOF MS spectrometry has been recognized to be a useful technique for determining the molecular weights of different saccharides and glycoconjugates, the sensitivity and the detectable mass range of saccharides are low compared to other biological polymers. The low detection sensitivity of saccharides is due to the fact that neutral polysaccharide molecules are difficult to cationized with Na^+^ instead of being protonated [5]. 

Permethylation of glycan [8] results in the conversion of all hydrogen atoms to methyl groups and serves to render glycans hydrophobic which may increase the signal intensity in mass measurement. Permethylated carbohydrates are considerably more stable than native glycans and produce more information in tandem mass spectra for their linkage analysis [3]. In this study we describe mass based approaches for permethylation-derivatized glycans such as dextran, glucomannan, β-1,3-glucan and pullulan (hexosyl polymer); arabinoxylan, and xylan (pentosyl polymer); and arabinogalactan (mixed hexosyl and pentosyl polymer). Permethylation derivatization is a common tool for mass spectrometry analysis of glycans and it is capable of improving MS sensitivity and stabilizing saccharides as well as glycuronic acids and sialic acids by converting the carboxylic groups into methyl esters [9,10,11]. However, permethylation involves complicated sample preparation (powdered sodium hydroxide or sodium hydride with methyl iodide in dimethyl sulfoxide) and clean up. In addition, the purification of permethylated glycans (with no chromophore) is difficult, especially for small amounts of sample.

Since most polysaccharides and permethylation-derivatized polysaccharides lack a chromophore, saccharides tagged with stable chemical groups at the reducing end are commonly used for glucan determination and purification [12]. For example, several different labels involve reductive amination labeling [12], and a number of reagents are commercially available for reductive amination reactions such as 2-aminobenzamide (2AB), 2-aminobenzoic acid (2AA) and 2-aminopyridine (2AP); 1-phenyl-3-methyl-5-pyrazolone (PMP) [13], and aldo-*bis*-indole (aldo-BIN) derivatives from indole labeling [14] are also used. Recently, we developed a new glycan tagging method [15,16] with 2,3-naphthalene diamine (NAIM) to label glycan at the reducing end to form a series of glycan-NAIM derivatives. These glycan-NAIMs provide higher intensities than the corresponding native sugars in MALDI-TOF MS analysis [17]. 

The imidazole ring is a class of heterocycles found in many biological compounds such as histidine and drugs. The 5-membered planar ring can serve as a base and as a weak acid in chemical and biological systems. One of the applications of imidazole is in the purification of His-tagged proteins through immobilized metal affinity chromatography (IMAC), which is used to elute tagged proteins bound to Ni^2+^ ions attached to the surface of beads in the chromatography. Benzimidazole has similar structure and function as imidazole that “fuses” benzene and imidazole. Benzimidazole and its derivatives can serve as a ligand for metal complexes, including vitamin B12 [18]. A new tagging method by OPD and histidine linked OPD (His-OPD) has been developed to form aldo-benzimidazole (aldo-BIM) and aldo-His-BIM derivatives and these tagged saccharides were applied for the improvement of MALDI-TOF MS analysis of polysaccharides [2,16]. We also demonstrated here that aldo-BIMs facilitated polysaccharide determination by incorporating the BIM moiety which plays a key role in increasing the ionization ability of glycan. Here, we used MALDI-TOF MS as a convenient tool to measure native polysaccharides and methylated or benzimidazole-derivatized glycans. 

## 2. Results and Discussion

### 2.1. MALDI*-*TOF MS of Native Polysaccharides

The investigation of non-derivatized oligosaccharides by MALDI-TOF MS with various matrices were reported [5,6,7,19]. DHB has been broadly used as a matrix in MALDI-TOF MS for neutral saccharides. Several types of glycan such as dextran (8,000 Da) with 2,5-DHB as matrix [20] and pullulan (5,000 Da) with THAP or 2,5-DHB as matrices [7,21] have been reported. In addition, we have also measured polysaccharides with a mass range of 2,000 Da on average from *Ganoderma lucidum*, which is well-known medicinal food with a β-(1,3)-β-(1,6)-D-glucan structure [22,23]. Commonly MALDI-TOF MS of saccharides need a high matrix-to-analyte ratio to give better signals. The matrix plays an important role in the ionization process for saccharides during the desorption process [24]. Native polysaccharides such as the soluble in cold water branched β-(1,3)-β-(1,6)-D-glucans (laminarans) from *S. cichorioides* and *Laminaria gurjanovae* are readily detected by MALDI-TOF MS using 2,5-DHB as a matrix in the molecular weight (MW) ranges from 2 to 7 kDa [25]. However, the linear β-(1,3)-D-glucan fraction from *L. gurjanovae*, hardly soluble in cold water, had a MW up to 25 kDa according to gel chromatography analysis and only up to 11 kDa using MALDI-TOF MS. Linear polysaccharides are probably more difficult to measure by MS because they require much harder conditions to ionize. 

**Figure 1:**
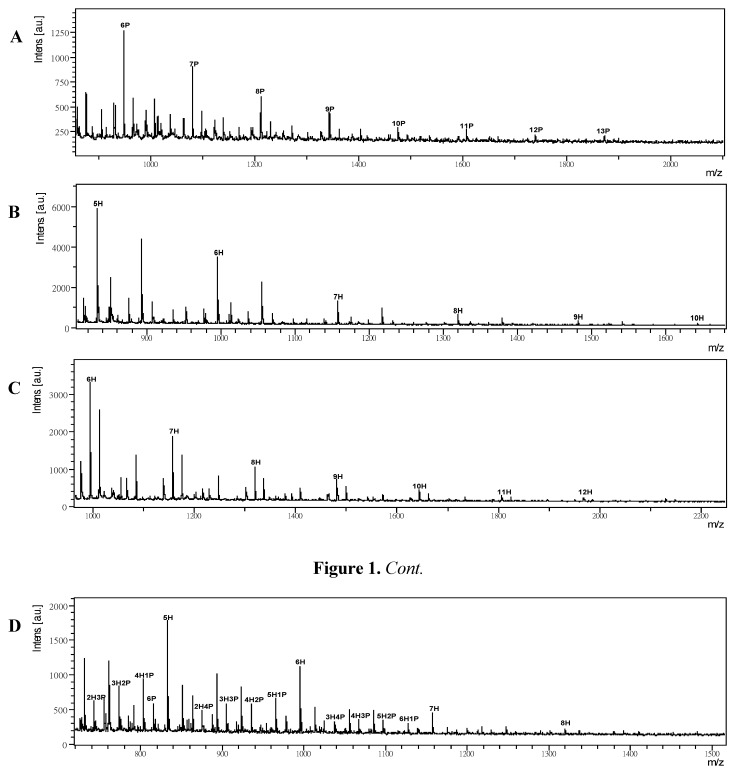
(**A**) MALDI-TOF MS spectrum of arabinoxylan. The mass difference of 132.1 Da between two neighboring peaks was observed. (**B**) The glucomannan spectrum with hexosyl units of 162.1 Da between two neighboring peaks is observed. (**C**) MALDI-TOF MS spectrum of dextran. (**D**) Mass spectrum of arabinogalactan with mixed pentose and hexose in polysaccharide. Experimental conditions: 2,5-DHB was used as matrix and the ratio of matrix to saccharide was 10:1. A Smartbeam 200 Hz solid-state laser was used. The sample load is set at 2 µL of 1,000 ppm polysaccharide solution in each dot on the steel plate.

The MALDI-TOF MS ionization efficiency for neutral oligosaccharides has been reviewed [26,27]. There are few native polysaccharides giving MALDI-TOF MS analysis without degradation and derivatization. Therefore, mass detection of native polysaccharides for the high mass ranges of glycan has not been reported. Only trace amounts of small saccharides are needed for detection in MS analysis. For example, Sturiale *et al*. resolved and identified Gram-negative bacteria through MALDI-TOF MS of native rough-type lipopolysaccharides (R-type LPSs) [28]. The samples were successfully and systematically adopted for the analysis of these complex biomolecules without prior chemical degradation. Here, we used commercial available polysaccharides with 2,5-DHB as matrix to study the improvement of signal intensities in mass spectrometry. We have also compared permethylated or BIM-derivatized polysaccharides. For example, the MALDI-TOF MS spectrum of arabinoxylan from 7P to 14P (where P represents pentose and number represents units) with mass peaks [nP–H_2_O+Na]^+^ at 947.5, 1079.6, 1211.6, 1343.7, 1475.8, 1607.8, 1739.9 and 1872.0 was observed (Figure 1A).

The peak-to-peak mass difference of 132.1 Da as one pentose is observed and native pentosyl polymers such as xylan show similar results. The masses range is set at 1 to 5 kDa for detectable polysaccharides by MALDI-TOF MS and the dispersive molecular masses with sequent gaps are the general property of natural glycan. For samples with higher masses it is increasingly difficult to get good spectra and the MALDI-TOF MS process is known to produce predominately singly charged ions with sodium attached. On the other hand, a similar observation was found in glucomannan (Figure 1B) and other hexosyl polymers such as dextran (Figure 1C), pullulan, larminarin and β-1,3-glucans. The glucomannan shows masses from 5H to 11H (H represents hexose and number represents units) as hexosyl units with mass peaks [nH–H_2_O+Na]^+^ at 833.3, 995.4, 1157.4, 1319.5, 1481.5, 1643.6 and 1805.6 were observed. The peak-to-peak mass difference of 162.1 Da was observed. Here, we also investigated a complicated polysaccharide like arabinogalactan which is built of a mixture of pentose and hexose (Figure 1D). The mass spectrum of arabinogalactan showed a hexosyl series (from 4H to 8H) and a pentose linked hexosyl saccharides series (e.g., 7H1P, 6H2P, 6H1P, 5H2P, 4H1P, *etc*.). The pentose is the subunits upon the hexosyl backone in arabinogalactan [29].

In recent years, MALDI-TOF MS has become a powerful tool for the determination of molecular weights of polymers. Other techniques like electrospray ionization are known to generate multiple charged ions and provide complex spectra due to a superposition of mass and charge distribution. Because of the huge mass range of polysaccharides MALDI-TOF MS is more suitable for the determination of molar mass distributions. There are some parameters that can influence mass spectra such as the voltage on the sample plate, the laser power and the choice of the matrix. 

### 2.2. MALDI-TOF MS of Permethylated Polysaccharides

**Figure 2:**
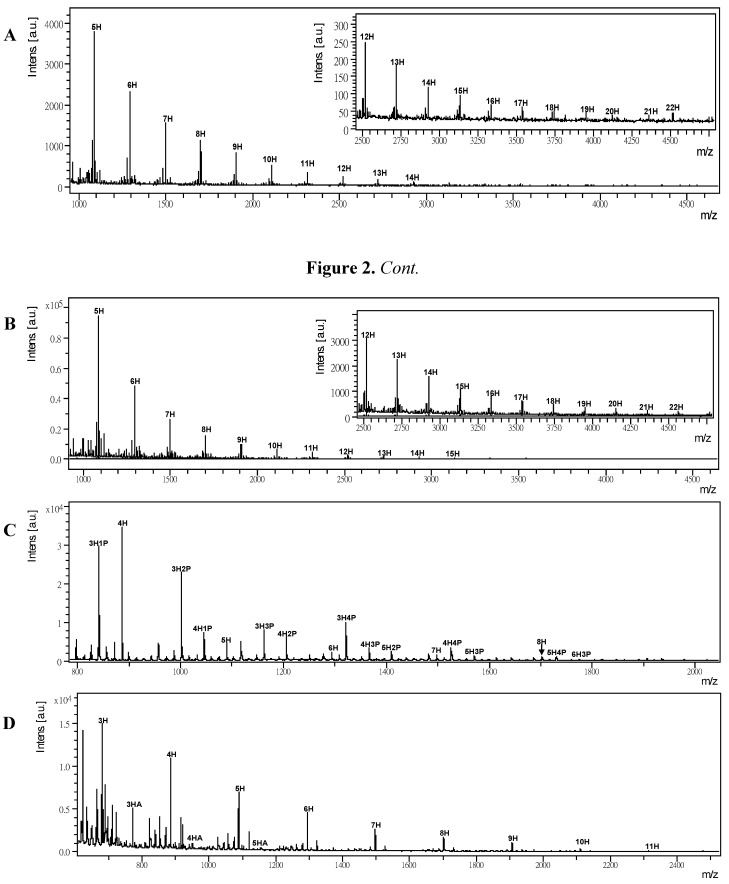
(**A**) MALDI-TOF MSspectrum of permethylated glucomannan. (**B**) MALDI-TOFMS spectrum of permethylated dextran (DP = 5 to 22). (**C**) MALDI-TOF MS spectrum of permethylated arabinogalactan. (**D**) MALDI-TOF MS of permethylated alginic acid. The conditions of MALDI-TOF MS are the same as that described in Figure 1.

Permethylation is a common method for the mass spectral analysis of glycans. This approach requires methylation of all the hydroxyl groups on saccharides. Permethylation is also useful for in-depth analysis of glycans as it provides information on linkages and branching that supplement GC-MS and tandem mass spectrometry for structural determination. Strategies for acquisition and interpretation of multistage MS have been fully developed for permethylated glycans [30]. For example, Hung *et al*. measured permethylated *G. lucidum* glucans using 2,5-DHB as a matrix [8,10,22]. Here, we investigated some permethylated polysaccharides such as arabinoxylan, xylan, glucomannan, dextran, β-1,3-glucans, arabinogalactan and alginic acids. For examples, Figure 2A shows the permethylated glucomannan that was observed as sodium attached ions and the masses for each peak can be calculated as [219.1 (a terminal sugar) + n × 204.1 (3 methylated hexosyl unit) + 31.0 (mass of OMe at reducing end residue) + Na]+ Da. The MALDI-TOF MS spectrum of permethylated glucomannan and dextran from 5H to 22H with mass ions [*P*nH+Na]^+^ at 1089.7, 1293.8, 1497.9, 1702.1, 1906.2, 2110.3, 2314.5, 2518.6, 2722.8, 2927.0, 3131.2, 3335.4, 3539.6, 3743.8, 3948.0, 4152.2, 4356.4 and 4560.6, while *P* represents permethylation, and a mass gap between two neighboring peaks was observed as 204.1 Da. The experiments on dextran (Figure 2B), pullulan, larminarin and β-1,3-glucans show similar results. The spectrum of the more complicated permethylated arabinogalactan was also measured (Figure 2C). It showed a hexosyl series (from *P*4H to *P*8H, *P* represents permethylation) and a pentose linked hexosyl saccharides series (e.g., *P*7H1P, *P*6H1P, *P*5H2P, *P*4H3P, *P*4H2P, *etc*.). The result implied that hexose is the major block in arabinogalactan [28]. In addition, polysaccharides from alginic acid, which is a kind of polysaccharide with a mixture of hexose and aldouronic acid was derivatized by NaOH or MeI in DMSO to get permethylated alginic acids. Their mass distributions were subsequently determined by MALDI-TOF MS (Figure 2D). The masses of permethylated alginic acids, which is composed of hexose units as sodium attached ions are at 681.4, 885.6, 1089.7, 1293.8, 1497.9, 1702.1, 1906.2, 2110.3 and 2314.5 (DP = 3 to 11), and a series of aldouronic acid at 723.5, 941.6 and 1159.8 (DP = 3 to 5) were determined. The peak-to-peak mass differences observed in the two series are 204.1 Da (three methyl hexosyl units) and 218.2 Da (three methyl hexosyl + 14; HexA unit), respectively. HexA belongs to the uronic acids it is a sugar acid with both a carbonyl and a carboxylic acid function. It is a hexose in which the C6 terminal carbon's hydroxyl function has been oxidized to a carboxylic acid.

In general, permethylation improves MS sensitivity in comparison of native polysaccharides, e.g., dextran 5H to 13H (Figure 1C) versus *P*5H to *P*22H (Figure 2B) were measured. Not only the detectable mass range was extended but also the signal intensities were stronger when the native polysaccharides and permethylated polysaccharides were compared. Polysaccharides with pentoses and hexoses including glycuronic acids, whose carboxylic groups are converted into methyl esters are capable of being converted into permethylated derivatives. However, permethylation involves complicated sample preparation and clean up with liquid-liquid extraction. Moreover, to purify non-chromophore permethylated glycans is quite difficult for small quantities of samples.

### 2.3. MALDI-TOF MS of Polysaccharide*-*BIMs

**Scheme 1:**
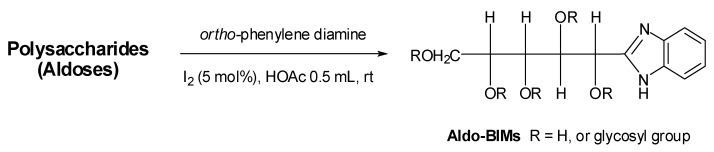
The preparation ofaldo-BIMs byiodine catalytic aldose-OPD condensation reaction.

**Figure 3:**
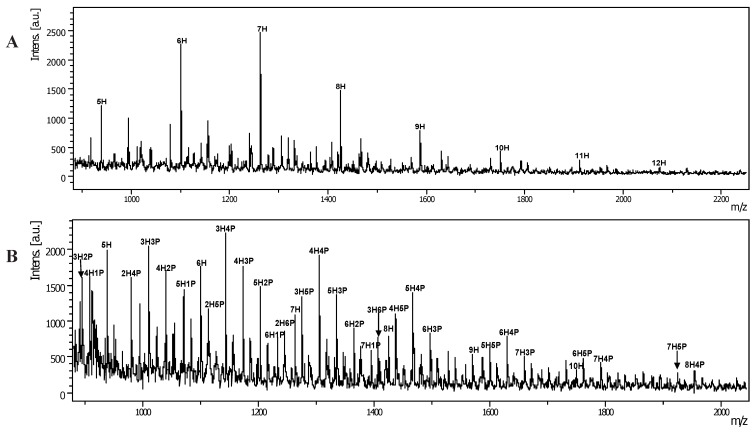
(**A**) MALDI-TOF MS of glucomannan-BIM derivatives. The mass difference of 162.1 Da was observed; (**B**) MALDI-TOF MS of arabinogalactan-BIM derivatives. The sample load is set at 2 µL of 1,000 ppm polysaccharide solution in each dot on the steel plate and the conditions of MALDI-TOF MS are the same as that described in Figure 1.

In a preliminary study, we used molecular iodine as catalyst to condense aldoses with aromatic *ortho*-diamines [15,16,17]. Here, we used OPD as tag to react with glycan (Scheme 1). This reaction is feasible for polysaccharide labeling of samples of less than 1 mg and the crude polysaccharide-BIM derivatives were precipitated by ethyl acetate. The residue was measured by MALDI-TOF MS directly without further purification or treatment. For example, glucomannan-BIMs were observed as sodium attached ions and the masses were calculated as [n × 162.1 (hexosyl unit) + 150.0 (reducing end residue) + 117.0 (1*H*-benzimidazole) + Na]^+^ Da, respectively. For instance, the MALDI-TOF MS spectrum of glucomannan–BIMs from 5H-BIM to 12H-BIM (where BIM represents the benzimidazole moiety) with the masses of these ion [nH–BIM+Na]^+^ at 939.5, 1101.5, 1263.6, 1425.7, 1587.8, 1749.9, 1911.9 and 2074.2 Da, respectively (Figure 3A), and a mass difference of 162.1 Da between neighboring peaks was observed. The experimental results for polysaccharides of dextran, pullulan, larminarin and β-1,3-glucans are similar. In addition, more complicated glycans such as arabinogalactan-BIM derivatives (Figure 3B) was also measured. The mass spectrum of arabinogalactan-BIM showed a hexosyl series (from 4H-BIM to 8H-BIM) and a pentose linked hexosyl saccharides series (e.g., 7H1P-BIN, 6H1P-BIM, 5H2P-BIM, 4H3P-BIM, 4H2P-BIM, *etc*.). In comparison the signals of arabinogalactan between underivatized glycan (Figure 1D), permethylated glycan (Figure 2C) and BIM tagged glycan (Figure 3B) show significant differences in molecular weights and intensities in the mass spectra. The signal intensities of permethylated glycans are higher than those in the corresponding underivatized or BIM-derivatized glycans. Moreover, other polysaccharides are amenable to BIM modification and determination by MALDI-TOF MS is in progress.

Not only glycan-BIM derivatives show increased intensities for high molecular weights compared to those of the native polysaccharides in MALDI-TOF MS measurements, but another advantage of this approach is that glycan-BIMs carry an UV active benzimidazole which could be used as a marker in chromatographic systems. The presence of an imidazole group on glycan could be useful as a base or nucleophile involving the 1*H*-amino group for advanced applications, for example, attachment of glycan-BIMs to solid supports for binding assays. In addition, glycan-BIM can possibly be used to differentiate native and derivatized glycan ions in MS measurements since the reducing end has a benzimidazole tag. 

## 3. Experimental

### 3.1. Chemicals and Reagents

Glucomannan, arabinoxylan, xylan, alginic acids, pullulan and larminarin were purchased from Megazyme (Wicklow, Ireland). Dextran, 2,5-DHB were purchased from Sigma-Aldrich (St. Louis, MO, USA). High purity β-1,3-glucan extracts from *Ganoderma lucidum* and *Saccharomyces pastorianus* were provided from Wyntek Corporation (Taipei, Taiwan) and Taiwan Tobacco & Liquor Corporation (Taipei, Taiwan). *Ortho*-phenylene diamine (OPD), 3,4*-*diaminobenzoic acid, methyl sulfoxide (DMSO), methyl iodide (MeI), acetic acid (AcOH) and iodine (I_2_) were purchased from Acros (Fair Lawn, NJ, USA). Sodium hydroxide (NaOH) was obtained from Merck (Darmstadt, Germany). Maltohexose was purchased from Supelco (Bellefonte, PA, USA). All materials are analytical grade and used without further purification. Milli-Q water (Millipore, Bedford, OH, USA) was used for the preparation of buffers and related aqueous solution. 

### 3.2. MALDI*-*TOF MS Measurement

MALDI-TOF MS experiments were performed on an UltraFlex II (Bruker Daltonics, Waldbronn, Germany) instrument operating in positive reflectron mode in the *m/z* range of 400–6,000. The spectra were acquired using the FlexControl software. A solid-state laser (Nd: YAG, 532 nm, SmartBeam, Waldbronn, Germany) with a frequency of 50 Hz was used for ionization. The samples of native and derivatized saccharide (1 mg/H_2_O solution; 1,000 ppm) with a fixed amount (100 nmol) ofmatrix of 2,5-DHB was loaded (2.0 µL) by a “dried-droplet” method or vacuum drying process. The accelerating voltage was set at 20 kV in positive ion mode. Typically, spectra were obtained by accumulating 500 laser shots for quantification. Laser energy per pulse was calibrated with a laser power meter (PEM 101, Berlin, Germany) so that laser fluence could be precisely measured. The delayed extraction time was adjusted from 100 ns to 500 ns. The grid voltage was set up as 95% of the accelerating voltage; the guide wire voltage was 0.2% of the accelerating voltage. The laser beam diameter was measured as ~100 μm on the sample target. The laser fluence was in the range of 50–300 mJ/cm^2^. The pressure inside of the flight tube was always kept between 10^−7^–10^−6^ Torr. 

### 3.3. Preparation of Permethylated Polysaccharide

In brief, the desiccated polysaccharide samples were dissolved in a DMSO suspension, which was prepared by vortexing DMSO and powdered NaOH or sodium hydride (NaH) at room temperature, an excess of MeI was added, and the solution was kept for several hours at room temperature with occasional vortexing. After finishing the reaction, the sample was extracted by adding chloroform in diluted aqueous AcOH solution, and the chloroform layer was concentrated. The sample was stored at −20 °C prior to analysis.

### 3.4. Preparation of Polysaccharide*-*BIMs

A suspension of polysaccharide (1.0 mg) in AcOH/H_2_O (v/v = 10:1, 1.0 mL; 1,000 ppm in solution) was treated with an *ortho*-phenylene diamine tag (1.0 mg, 10 μmol) and iodine (0.1 mg, 0.5 μmol in AcOH). The reaction mixture was stirred at room temperature for 12 h as indicated by the thin layer chromatography (TLC) analysis. The mixture was precipitated with EtOAc and organic layer was removed, and the residue was dried in vacuum to give the crude product of glycan-BIMs. These glycan-BIM derivatives were directly determined by MALDI-TOF MS without further purification. 

## 4. Conclusions

We have used MALDI-TOF MS for polysaccharide determination. Various types of native polysaccharides and permethylated or benzimidazole-derivatized glycans were analyzed to compare their signal intensity in MALDI-TOF MS. In contrast to the parent native polysaccharides, the permethylated polysaccharides have the strongest intensities in mass measurement. The glycan-BIM derivatives which containing chromophore showed both signal enhancement and visibility in MS and purification processes. In comparison with other reducing end tagging methods, the production of glycan-BIM is easier and they can enhance the signals of saccharide. We report here a benzimidazole moiety as a novel tag to attach to glycans for MALDI-TOF MS measurement of polysaccharides. This method is promising in application to measure/enrich tiny amounts of glycan in samples. 
